# Epidermal Growth Factor Receptors in Vascular Endothelial Cells Contribute to Functional Hyperemia in the Brain

**DOI:** 10.3390/ijms242216284

**Published:** 2023-11-14

**Authors:** Hannah R. Ferris, Nathan C. Stine, David C. Hill-Eubanks, Mark T. Nelson, George C. Wellman, Masayo Koide

**Affiliations:** 1 Department of Pharmacology, Larner College of Medicine University of Vermont, Burlington, VT 05405, USA; hannah.ferris@cuanschutz.edu (H.R.F.); nathan.stine@uvm.edu (N.C.S.);; 2 Vermont Center for Cardiovascular and Brain Health, Larner College of Medicine University of Vermont, Burlington, VT 05405, USA; 3 Division of Cardiovascular Sciences, University of Manchester, Manchester M13 9PL, UK

**Keywords:** functional hyperemia, epidermal growth factor receptor (EGFR), vascular endothelial cells, phosphatidylinositol 4,5-bisphosphate (PIP_2_), cerebral small vessel diseases (cSVD)

## Abstract

Functional hyperemia—activity-dependent increases in local blood perfusion—underlies the on-demand delivery of blood to regions of enhanced neuronal activity, a process that is crucial for brain health. Importantly, functional hyperemia deficits have been linked to multiple dementia risk factors, including aging, chronic hypertension, and cerebral small vessel disease (cSVD). We previously reported crippled functional hyperemia in a mouse model of genetic cSVD that was likely caused by depletion of phosphatidylinositol 4,5-bisphosphate (PIP_2_) in capillary endothelial cells (EC) downstream of impaired epidermal growth factor receptor (EGFR) signaling. Here, using EC-specific EGFR-knockout (KO) mice, we directly examined the role of endothelial EGFR signaling in functional hyperemia, assessed by measuring increases in cerebral blood flow in response to contralateral whisker stimulation using laser Doppler flowmetry. Molecular characterizations showed that EGFR expression was dramatically decreased in freshly isolated capillaries from EC-EGFR-KO mice, as expected. Notably, whisker stimulation-induced functional hyperemia was significantly impaired in these mice, an effect that was rescued by administration of PIP_2_, but not by the EGFR ligand, HB-EGF. These data suggest that the deletion of the EGFR specifically in ECs attenuates functional hyperemia, likely via depleting PIP_2_ and subsequently incapacitating Kir2.1 channel functionality in capillary ECs. Thus, our study underscores the role of endothelial EGFR signaling in functional hyperemia of the brain.

## 1. Introduction

Blood flow within the brain alters dynamically in response to spatial and temporal changes in neuronal activity, redirecting the delivery of blood-borne nutrients so as to support the elevated metabolic demands of active neurons. This phenomenon, termed functional hyperemia, is essential for maintaining brain health and computational activities, including cognition [1,2,3,4]. Functional hyperemia is mediated by an ensemble of neurovascular coupling mechanisms that translate enhancements in neuronal activity to increases in local blood flow through the integrated activity of multiple cell types of the neurovascular unit, comprising (in addition to neurons) astrocytes, vascular endothelial cells (ECs), vascular smooth muscle cells (SMCs), and pericytes [2,5,6,7,8]. Accumulating evidence has indicated that, among these cell types, brain capillary ECs, which are the building blocks of the vast, anastomosing network of capillaries—the smallest and most abundant vessels in the brain parenchyma—play a key role in linking neuronal activity to upstream arteriolar dilation [8,9,10]. Importantly, our recent study demonstrated that capillary EC inward-rectifier Kir2.1 channel is a critical player in the initiation of this inter-cellular communication [8]. The Kir2.1 channel initiates the vasodilatory signal by sensing focal neuronal activity in the form of modest increases in perivascular potassium ion (K^+^) concentrations caused by K^+^ efflux during each neuronal action potential. Increased levels of perivascular K^+^, in turn, activate capillary EC Kir2.1 channels, which cause EC membrane potential hyperpolarization. Given that Kir2.1 channels are also activated by membrane potential hyperpolarization, Kir2.1 channels in adjacent capillary ECs are subsequently activated, resulting in propagation of the original hyperpolarizing (i.e., electrical) signal from EC to EC within the capillary bed. These signals ultimately reach upstream arterioles, where they hyperpolarize overlying SMCs, causing arteriolar/artery dilation through a decrease in the open-state probability of voltage-dependent Ca^2+^ channels—a primary source of Ca^2+^ influx in vascular SMCs. This upstream arteriolar dilation results in increased local blood flow to the point of signal origin (i.e., region of heightened neuronal activity). Thus, capillary EC Kir2.1 channel activation is a keystone of neuronally triggered vasodilation, which is crucial for functional hyperemia in the brain [8,11].

In addition to being regulated by membrane potential and extracellular K^+^ concentration, Kir2.1 channel activity is also modulated by the plasma membrane phospholipid, phosphatidylinositol 4,5-bisphosphate (PIP_2_) [12,13]. PIP_2_ is a requisite co-factor for Kir2.1 activity; thus, under PIP_2_-deficient conditions, such as in the context of increased PIP_2_ hydrolysis by Gq-type G-protein–coupled receptor (GqPCR) signaling-induced phospholipase C (PLC) activation, Kir2.1 channel activity is decreased [14]. Consistent with the idea that Kir2.1 channel activation is a critical step in capillary-initiated vasodilation, the attendant PIP_2_ depletion caused by activation of PLC through systemic administration of the GqPCR agonist, carbachol, decreases Kir2.1 currents in capillary ECs and attenuates capillary Kir2.1-mediated capillary-to-arteriole vasodilatory responses ex vivo and in vivo [14]. Notably, a PIP_2_ deficiency in capillary ECs has been shown to contribute to functional hyperemia deficits in a mouse model of CADASIL (cerebral autosomal dominant arteriopathy with subcortical infarcts and leukoencephalopathy)—the most common monogenic inherited form of cerebral small vessel diseases (cSVD) [15]. Strikingly, exogenous administration of PIP_2_ in CADASIL model mice rescues these deficits in capillary Kir2.1 channel currents, capillary-initiated arteriolar dilation, and functional hyperemia [15].

CADASIL is caused by mutations in the extracellular domain of the *NOTCH3* gene [16,17], the pathology of which is characterized by small vessel dysfunction in the brain and ultimately leads to cognitive decline [18,19,20]. Notch3 is a single transmembrane protein [21] that is predominantly expressed in SMCs and pericytes in the brain vasculature [22,23]. Mutated NOTCH3 ectodomain (Notch3^ECD^) interacts with extracellular matrix proteins and forms granular osmiophilic materials (GOM) deposition within the perivascular space of brain parenchyma [24,25,26]. Previous studies have demonstrated that NOTCH3^ECD^-containing perivascular GOM deposits, a hallmark of CADASIL, involve accumulations of the matrix metalloproteinase inhibitor, TIMP3 [26,27,28], and its downstream signaling plays a pivotal role in the vascular pathologies of the disease [15,29,30,31]. That is, TIMP3 suppresses the activity of the matrix metalloproteinase, ADAM17, and prevents shedding/maturing of the epidermal growth factor receptor (EGFR) ligand, heparin-binding EGF-like growth factor (HB-EGF), resulting in decreased EGFR activity [15,29,30]. Importantly, *NOTCH3* gene mutation attenuates EGFR signaling not only in SMC-containing arterioles but also in pericyte-adjacent capillary ECs, which are also in contact with GOM deposits. Indeed, brain capillary ECs isolated from CADASIL model mice exhibit significantly diminished Kir2.1 currents that can be rescued by exogenous HB-EGF. In addition, HB-EGF rescues capillary-to-arteriole vasodilatory signaling and functional hyperemia in CADASIL mice—effects comparable to those produced by PIP_2_ administration [15]. These findings suggest that down-regulated capillary EC EGFR signaling promotes a reduction in plasma membrane PIP_2_ levels, in turn decreasing Kir2.1 channel activity, crippling capillary-initiated vasodilatory responses and impairing functional hyperemia in CADASIL.

Here, using newly developed EC-EGFR-knockout (KO) mice, we sought to elucidate the role of endothelial EGFR signaling in functional hyperemia. To this end, we evaluated functional hyperemia, measured as an increase in cerebral blood flow (CBF) in the barrel cortex in response to contralateral whisker stimulation, using laser Doppler flowmetry. We further tested whether treatment with the Kir2.1 channel co-factor PIP_2_ or the EGFR ligand HB-EGF altered functional hyperemia in EC-EGFR-KO mice.

## 2. Results

### 2.1. Characterization of Capillary ECs from EC-EGFR-KO Mice

**Figure 1:**
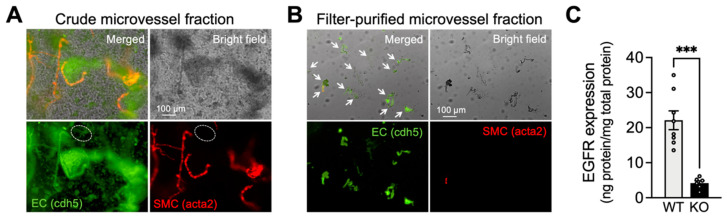
Characterization of capillary ECs from EC-EGFR-KO mice. (**A**,**B**): Bright field and fluorescent images of isolated microvessels from EC/SMC dual-reporter mice before (**A**) and after two-step filter purification (**B**). Green fluorescent protein expresses in ECs under the cadherin-5 (cdh5) promotor, and red fluorescent protein expresses in SMC/pericytes under the acta2 promoter. The vast majority (>98%, counted by cell numbers after DAPI staining) of tissue obtained by two-step filter purification was green fluorescence-positive and red fluorescence-negative microvessels, demonstrating that the collected tissue samples were capillaries. White arrows indicate microvessels in filter-purified microvessel fraction (panel **B**). In contrast, the crude microvessel fraction (before the two-step filter-purification) contains red fluorescence-positive microvessels (i.e., arterioles). Note: The green fluorescent image of crude microvessel fraction also shows auto-fluorescence in elastic lamina membranes and brain tissue debris. An example of green fluorescence-positive and red fluorescence-negative microvessels (i.e., capillaries) in crude microvessel fractions are circled by white dotted lines. Experiments were repeated four times and exhibited consistent and similar results. (**C**): EGFR protein in freshly isolated capillaries, pooled from 4 animals for each sample, were quantified by ELISA. EGFR protein was dramatically decreased in EC-EGFR-KO mice compared to WT animals (i.e., Cre-negative littermates). Data are presented as mean ± SEM (*n* = 8 samples from 32 WT animals, *n* = 6 samples from 24 KO mice). *** *p* < 0.001 between groups by unpaired *t*-test.

To characterize the newly developed inducible EC-EGFR-KO mouse model, we first confirmed ablation of EGFR protein in brain capillary ECs of these mice. Capillaries were isolated and collected from the brain cortex using a modification of a previously described protocol [32], as detailed in Methods. The purity of isolated capillary samples was verified using a dual-promoter double-color (acta2-RCaMP/cdh5-GCaMP8) mouse strain, which expresses the red fluorescent protein (RCaMP) in SMCs and pericytes, and the green fluorescent protein (GCaMP8) in ECs. As shown in Figure 1A,B, the vast majority (98.1 ± 1.1%, *n* = 4 trials using 4 separate animals) of filter-purified microvessels obtained from these EC/SMC dual-reporter mice were GCaMP-positive and RCaMP-negative, demonstrating that these samples contained capillary ECs and were largely devoid of SMCs. Next, using freshly isolated capillary ECs samples from EC-EGFR-KO and WT mice, we quantified the EGFR protein using a sandwich enzyme-linked immunosorbent assay (ELISA). Total EGFR protein expression in capillary ECs was dramatically (~five-fold) decreased in EC-EGFR-KO mice (4.2 ± 0.7 ng/mg; *n* = 6 assay trials using a total of 24 mice) compared with that in wild-type (WT), Cre-negative littermates (22.1 ± 2.7 ng/mg; *n* = 8 assay trials using a total of 32 mice) (Figure 1C). No differences in body weight, blood pressure, or heart rate were observed between EC-EGFR-KO and WT animals (Table 1), consistent with previously reported data from a strain of EC-EGFR-KO mice generated using the non-inducible Tie2-promoter [33].

### 2.2. Impaired Functional Hyperemia in EC-EGFR-KO Mice

**Figure 2:**
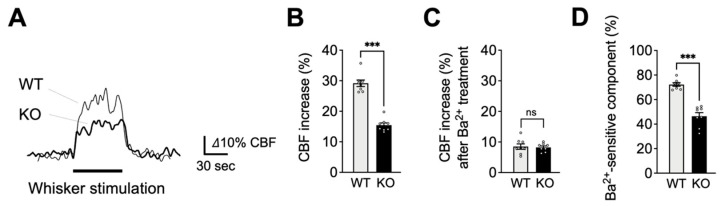
Impaired functional hyperemia in EC-EGFR-KO mice. (**A**): Representative traces showing CBF increases in contralateral somatosensory cortex during whisker stimulation. Cre-negative littermates (WT) were used as a control group in comparison to EC-EGFR-KO (KO) mice. (**B**): Summary data of whisker stimulation-induced functional hyperemia. (**C**): CBF increase during whisker stimulation after treatment with Ba^2+^ (100 μM), a Kir2.1 channel blocker. (**D**): Ba^2+^-sensitive component of functional hyperemia, i.e., the difference in responses before and after Ba^2+^ treatment, indicating the contribution of the Kir2.1 channels to whisker stimulation-induced functional hyperemia. Data are presented as mean ± SEM (*n* = 8 animals in WT, *n* = 8 animals in KO). *** *p* < 0.001, ns: not significant between groups by unpaired *t*-test.

We next assessed functional hyperemia in EC-EGFR-KO and WT mice using laser Doppler flowmetry. As shown in Figure 2A,B, contralateral whisker stimulation caused a 29.2% ± 1.1% increase in CBF in WT animals (*n* = 8); by comparison, the same stimulation caused a significantly attenuated (~50%) hyperemic response (15.4 ± 0.7% increase) in EC-EGFR-KO mice (*n* = 8). To evaluate the contribution of Kir2.1 channel-mediated signaling, we topically applied the selective Kir2.1 channel blocker, Ba^2+^ (100 μM), to the superfusate flowing over the somatosensory cortex [8,15,34]. Consistent with the previously established involvement of Kir2.1 channels in the functional hyperemic response to whisker stimulation [8,15,34], this treatment substantially decreased functional hyperemia in both EC-EGFR-KO and WT mice, leaving the same residual response in both genotypes (Figure 2C). However, the Kir2.1-mediated (i.e., Ba^2+^-sensitive) component of this reduction was greater in WT mice (70.9% ± 2.5% reduction; *n* = 8) than in EC-EGFR-KO mice (46.8% ± 2.9% reduction; *n* = 8) (Figure 2D), suggesting that EC-specific EGFR ablation eliminated much of the Kir2.1-dependent response. Collectively, these observations indicate that ablation of EC-EGFR impairs Kir2.1-mediated capillary-to-arteriole vasodilatory signaling.

### 2.3. PIP_2_, but Not HB-EGF, Restores Functional Hyperemia in EC-EGFR-KO Mice

**Figure 3:**
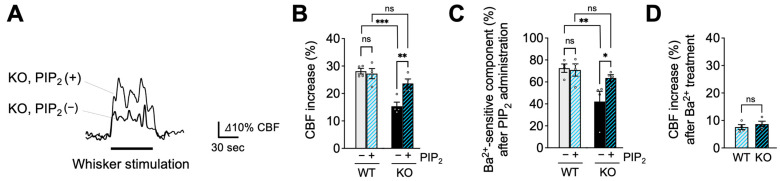
PIP_2_, an endogenous Kir2.1 channel co-factor, restored functional hyperemia in EC-EGFR-KO mice. (**A**): Whisker stimulation-induced functional hyperemia before and after PIP_2_ treatment in EC-EGFR-KO mice. (**B**): Summary data showing PIP_2_ treatment restores functional hyperemia deficits in EC-EGFR-KO mice, with little impact on WT animals. (**C**): Ba^2+^-sensitive component of functional hyperemia before and after PIP_2_ treatment. (**D**): CBF increase during whisker stimulation after Ba^2+^ treatment. Ba^2+^ was cortically superfused for 20 min following PIP_2_ treatment. Data are presented as mean ± SEM (*n* = 4 animals in WT, *n* = 4 animals in KO). *** *p* < 0.001, ** *p* < 0.01, * *p* < 0.05, ns: not significant between groups by two-way ANOVA (**B**,**C**) or by unpaired *t*-test (**D**).

To further explore the basis of Kir2.1 channel impairment in EC-EGFR-KO mice, we examined the ability of PIP_2_, an endogenous Kir2.1 co-factor, to reverse functional hyperemia deficits in these mice. To this end, we administered the synthetic PIP_2_ analog, dipalmitoyl-PIP_2_ (0.5 mg/kg body weight), to EC-EGFR-KO mice or WT mice through a catheter placed in the femoral artery [15] and tested whisker stimulation-induced functional hyperemia 20 min later. As shown in Figure 3A,B, this maneuver significantly increased functional hyperemic responses in EC-EGFR-KO mice (23.6% ± 1.6% increase in CBF; *n* = 4) compared with those elicited prior to PIP_2_ administration (15.3% ± 0.7% increase in CBF; *n* = 4). Consistent with previous observations [15], PIP_2_ administration did not impact functional hyperemic responses to whisker stimulation in WT animals (28.1% ± 1.0% and 27.2 ± 1.8% before and after PIP_2_, respectively; *n* = 4) (Figure 3B). We next examined the impact of PIP_2_ administration on Kir2.1-mediated signaling (i.e., Ba^2+^-sensitive component of functional hyperemia) via subsequent treatment of the selective Kir2.1 channel blocker, Ba^2+^. Interestingly, the Kir2.1-mediated (i.e., Ba^2+^-sensitive) component in EC-EGFR-KO mice was increased by PIP_2_ treatment to a level comparable to that observed in WT mice (Figure 3C). The residual responses after cortical application of Ba^2+^ were similar in both genotypes (Figure 3D). Thus, PIP_2_ administration enhanced the Kir2.1-mediated component in EC-EGFR-KO mice, indicating that PIP_2_ supplementation improved Kir2.1 channel function.

**Figure 4:**
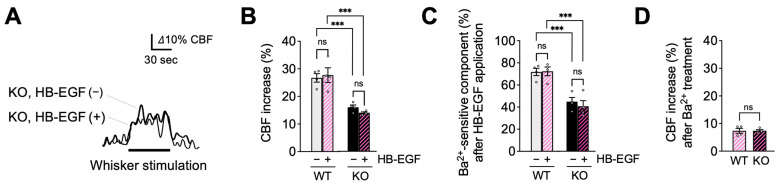
HB-EGF, an EGFR ligand, failed to restore functional hyperemia in EC-EGFR-KO mice. (**A**): Whisker stimulation-induced functional hyperemia before and after HB-EGF treatment in EC-EGFR-KO mice. (**B**): Summary data showing that HB-EGF treatment did not alter whisker stimulation-induced functional hyperemia in either EC-EGFR-KO or WT mice. (**C**): Ba^2+^-sensitive component of functional hyperemia before and after HB-EGF treatment. (**D**): Whisker stimulation-induced CBF increase after Ba^2+^ treatment. Ba^2+^ was subsequently and concurrently applied for 20 min by adding to HB-EGF-containing cortical superfusate. Data are presented as mean ± SEM (*n* = 4 animals in WT, *n* = 4 animals in KO). *** *p* < 0.001, ns: not significant between groups by two-way ANOVA (**B**,**C**) or by unpaired *t*-test (**C**).

In the following experimental series, we tested whether treatment with the EGFR ligand, HB-EGF, which rescues functional hyperemic responses in CADASIL model mice [29,30], could similarly normalize functional hyperemia in EC-EGFR-KO mice. Consistent with the absence of its cognate receptor, HB-EGF (30 ng/mL), topically applied for 20 min [15,29], did not alter functional hyperemic responses in EC-EGFR-KO mice, which exhibited persistent functional hyperemia deficits (with HB-EGF, 14.0% ± 1.6%; without HB-EGF, 16.0% ± 0.8%; *n* = 4). The subsequent Ba^2+^ application revealed that the Ba^2+^-sensitive component also remained significantly smaller in EC-EGFR-KO mice (Figure 4C,D). Functional hyperemia was also unaffected by HB-EGF treatment in WT animals, which presented similar increases in cortical blood flow in response to whisker stimulation before and after HB-EGF (with HB-EGF, 27.7% ± 2.6%; without HB-EGF, 26.7% ± 1.5%; *n* = 4). These data suggest that PIP_2_ depletion contributes to the decreased activity of capillary EC Kir2.1 channels and functional hyperemia deficits in EC-EGFR-KO mice.

## 3. Discussion

**Figure 5:**
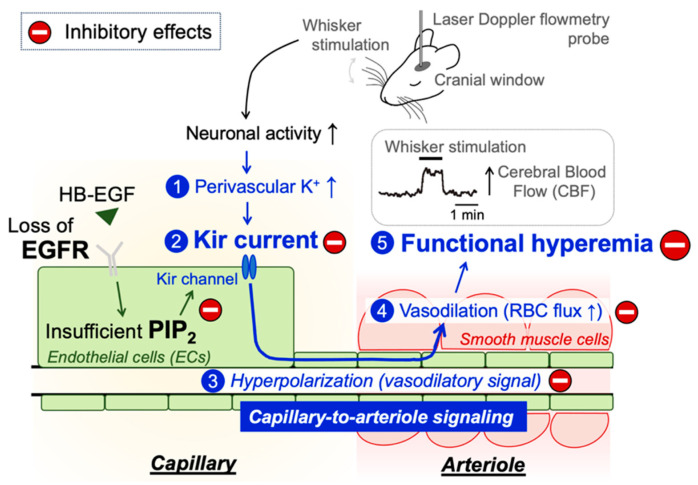
Schematic illustration of the proposed signaling pathway underlying functional hyperemia deficits in EC-EGFR-KO mice. Whisker stimulation and subsequent neuronal activation, which increases perivascular K^+^ during neuronal repolarization, results in Kir2.1 channel activation in capillary ECs. Kir2.1 channel-initiated hyperpolarizing vasodilatory signal rapidly propagates upstream and dilates the precapillary arteriole, increasing downstream tissue perfusion. This local increase in blood flow in neuronally active regions of the brain is termed functional hyperemia, which is detected using laser Doppler flowmetry in this study. Genetic deletion of EGFR disrupts functional hyperemia in response to whisker stimulation by causing Kir2.1 channel dysfunction, which is likely attributed to lowering the level of PIP_2_, an essential co-factor of Kir2.1 channels.

In this study, we examined the role of endothelial EGFR signaling in functional hyperemic responses in the brain using newly developed EC-specific EGFR-KO mice. EGFR, originally identified as a cancer-promoting protein [35], is now known to be involved in a multitude of (patho)physiological phenomena [36,37,38,39], including functional hyperemia in the brain [15,29]. Here, we validated the loss of EGFR protein in capillaries of EC-EGFR-KO mice and show that the selective ablation of endothelial EGFR significantly impairs functional hyperemia, likely via disrupting Kir2.1 channel-mediated capillary-to-arteriole signaling (Figure 5). These findings are consistent with our prior work using CADASIL model mice harboring mutations in the *NOTCH3* gene, which indicated that functional hyperemia is attenuated by EGFR-dependent down-regulation of Kir2.1 activity in ECs [15]. Importantly, in CADASIL mice, Kir2.1 channel dysfunction was observed only in capillary ECs among various cell types in the brain vasculature [15]. Moreover, functional hyperemia deficits in CADASIL mice were rescued by treatment with HB-EGF [15], an observation that suggested that disabled endothelial EGFR signaling is a major pathology in functional hyperemia deficits in CADASIL. Collectively with the current study using EC-EGFR-KO mice, these data confirm that endothelial EGFR signaling is an important contributor to functional hyperemia in the brain.

Functional hyperemia deficits in EC-EGFR-KO mice are likely attributable to crippled Kir2.1 channel-mediated signaling, as supported by the diminished Ba^2+^-sensitive component of whisker-stimulated increases in CBF in these mice (Figure 2D). This is consistent with a previous report that loss of Kir2.1 channel function (e.g., through genetic deletion of Kir2.1 channels in ECs [8]) leads to functional hyperemia deficits. In CADASIL model mice, Kir2.1 dysfunction and subsequent functional hyperemia deficits were shown to result from an insufficiency of the Kir2.1 channel co-factor, PIP_2_, and those were restored by supplemental treatment with exogenous PIP_2_ [15]. Similar to the case for CADASIL mice, this study showed that functional hyperemia deficits in EC-EGFR-KO mice were alleviated by the administration of PIP_2_ (Figure 3). These findings suggest that the elimination of endothelial EGFRs reduces PIP_2_ levels, subsequently impairing functional hyperemia by diminishing Kir2.1 channel activity. Further, the observations that exogenous PIP_2_ or HB-EGF did not cause additional increases in functional hyperemia response in WT mice may indicate that EGFR-intact animals hold sufficient PIP_2_ in their capillary EC plasma membrane to sustain Kir 2.1 channel functionality. These results are underpinned by previous findings that exogenous PIP_2_ or HB-EGF does not increase Kir2.1 channel currents in freshly isolated capillary ECs from wildtype animals [15], suggesting that Kir2.1 channels in capillary ECs in EGFR-intact animals have access to sufficient PIP_2_ at the plasma membrane. Interestingly, it has been reported that PIP_2_ treatment enhances Kir2.1 currents in arterial ECs [40] or Kir2.x channel currents in *Xenopus* oocyte expression systems [12], suggesting that basal PIP_2_ levels and/or PIP_2_-dependent regulation of Kir2.1 channel functionality may differ depending on cell types. Indeed, in CADASIL mice, Kir2.1 channel dysfunction is observed only in capillary ECs, and not in arteriolar SMCs, despite EGFR signaling being downregulated in both ECs and SMCs [15,29,30]. The detailed mechanism by which the EGFR maintains PIP_2_ levels in capillary ECs remains to be elucidated. However, this study demonstrates that endothelial EGFR signaling is critical to retain capillary Kir2.1 channel functionality and functional hyperemia response.

Our results indicate that the endothelial EGFR signaling is a requisite component of neuronal activity-dependent increases in local blood perfusion. However, this work does not exclude a contribution of smooth muscle EGFR signaling (which plays a role in arterial/arteriolar contractility) in functional hyperemia in the brain. In cerebral arteries [41] and arterioles [30], activation of smooth muscle EGFR by ligands, such as HB-EGF, promotes vasoconstriction via suppression of voltage-gated potassium (K_V_1.5) channel currents, leading to membrane depolarization and increased intracellular Ca^2+^ [30,42]. Thus, activation of smooth muscle EGFRs causes arterial constriction directly [30,43] and/or enhances agonist-induced contractile responses [44]. In CADASIL, which results from mutant Notch3^ECD^ accumulation-dependent dysregulation of TIMP3/ADAM17/HB-EGF/EGFR signaling, the decreased EGFR signaling is not limited to ECs but also impacts arterial SMCs. Hence, intraluminal pressure-induced cerebral artery constriction (myogenic tone), a mechanism intrinsic to SMCs, is greatly attenuated in CADASIL mice, which can be recovered by treatment with HB-EGF [30]. Therefore, it is conceivable that HB-EGF restores functional hyperemic responses in CADASIL mice by improving both endothelial EGFR signaling (through enhanced capillary Kir2.1 channel function), as well as smooth muscle EGFR signaling (by impacting arterial contractility and/or vasodilatory capability). Nonetheless, this present study clearly demonstrates that selective loss of EC EGFR signaling is sufficient to disrupt functional hyperemia, consistent with endothelial EGFR signaling playing a key role in the regulation of focal vasodilatory responses within active regions of the brain.

## 4. Material and Methods

### 4.1. Animals

EC-EGFR-KO mice were obtained by crossbreeding cdh5-CreERT2 mice (obtained from Dr. Ralf Adams, Cancer Research UK London Research Institute; University of Münster, Germany) [45] and floxed-EGFR (kindly gifted from Dr. David Thredgill, Texas A&M University) [46]. Cadherin-5 (cdh5, aka VE-cadherin) is a vascular endothelial cell-specific adhesion molecule and was employed as an EC-specific promoter [45]. Cdh5-CreERT2-positive, floxed-EGFR homozygous offspring, including both males and females, were treated with tamoxifen by adding it to their chow (~40 mg/kg body weight/day; Envigo, #TD.130859, Indianapolis, IN, USA) at 8 weeks of age for 7 days. Animals were used experimentally 2–4 weeks after ending tamoxifen treatment to allow for extinction of pre-existing EGFR protein. Cre-negative littermates that underwent identical tamoxifen treatment were used as control “wildtype (WT)” animals. EC/SMC dual-reporter mice were obtained by crossbreeding of cdh5-GCaMP8 (JAX strain #33342; CHROMus collaboration, The Jackson Laboratory, Bar Harbor, ME, USA) [47] and acta2-RCaMP (JAX strain #028345; CHROMus collaboration, The Jackson Laboratory, Bar Harbor, ME, USA) [47,48]. This dual-promoter double-color mouse strain has green fluorescent protein in cdh5-positive cells (i.e., endothelial cells) [47] and red fluorescent protein in acta2-positive cells (i.e., smooth muscle cells and pericytes [47]), and was used to distinguish these cell types. All experimental protocols used in this study complied with ARRIVE guidelines [49] and were approved by the Institutional Animal Care and Use Committee of the University of Vermont (protocol ID: X0-009).

### 4.2. Isolation of Brain Parenchymal Capillaries

Capillaries were collected from both cerebral cortices using a modified protocol that was previously described for collection of cerebral microvessels [32]. Briefly, pia membrane and brain surface vessels were physically removed from isolated cortices. Cortices were then homogenized in ice-cold artificial cerebrospinal fluid (aCSF: 125 mM NaCl, 3 mM KCl, 26 mM NaHCO_3_, 1.25 mM NaH_2_PO_4_, 1 mM MgCl_2_, 4 mM glucose, 2 mM CaCl_2_, pH 7.3) using a Dounce homogenizer and homogenates centrifuged at 2000× *g* for 5 min at 4 °C. Crude tissue precipitates were re-suspended in 17.5% dextran (~70 kDa, in aCSF) and centrifuged at 4000× *g* for 20 min at 4 °C, which allowed separation of a blood vessel-rich precipitate (crude microvessel fraction, Figure 1A) from an upper layer of myelin. Next, capillaries were isolated from blood vessel-rich precipitate via a two-step filtration process. Firstly, the vasculature-rich fraction was re-suspended in aCSF and filtered through a 200 μm mesh strainer, removing large vessels that were retained in the filter. The remaining suspension was then filtered through a 40 μm mesh strainer. Capillary-rich microvessels retained on the 40 μm mesh strainer were retrieved by washing off the inverted strainer with aCSF, and collected by centrifuging at 2000× *g* for 5 min. As detailed below, we characterized the collected microvessel tissue by the combined use of EC/SMC dual-reporter mice and epi-fluorescent microscopy. The vast majority (>98%) of tissue obtained by this method were capillaries, which lack smooth muscle cells (Figure 1B).

### 4.3. Epi-Fluorescent Microscopy

The brain microvessels collected by the method described above were examined by epi-fluorescent microscopy. Bright-field and fluorescent images of freshly isolated micro-vessels from EC/SMC dual-reporter mice were acquired by a Nikon Eclipse Ti epi-fluorescent microscope equipped with an Andor Clara high-resolution interline CCD camera, Nikon Ti LED illuminator, and NIS Elements 4.20 software. With these dual reporter mice, green fluorescent protein was expressed in ECs under the cadherin-5 (cdh5) promotor and red fluorescent protein was expressed in SMC/pericytes under the acta2 promoter. Images were obtained using the following fluorescent filter sets: Ex 460–500 nm/Em 510–560 nm for green and Ex 540–580 nm/Em 600–660 nm for red. The capillary population was evaluated as the percentage of green-fluorescent-positive, red-fluorescent-negative cell numbers against total cell numbers, counted using Image J 1.53 software (NIH, http://imagej.nih.gov/ij accessed on 11 November 2023) after DAPI staining.

### 4.4. Measurement of EGFR Expression

EGFR protein was quantified in cortical capillary preparations by ELISA (Invitrogen, EM23RB, obtained through ThermoFisher, Waltham, MA, USA) following the manufacturer’s instruction. Freshly isolated and collected capillaries, pooled from 4 animals for each sample, were lysed in 200 μL of T-PER™ tissue protein extraction reagent (Thermo-Fisher, Waltham, MA, USA) containing protease inhibitors (1 mM AEBSF, 0.8 μM aprotinin, 50 μM bestatin, 15 μM E-64, 5 mM EDTA, 20 μM leupeptin, 10 μM pepstatin A). Due to the low yield of capillaries using the collection method described above, we pooled the tissue from 4 animals into one sample to obtain an adequate amount of protein for duplicate measurement of EGFR expression and for protein assay. Samples (90 μL per well, in duplicates) or standard protein (mouse recombinant EGFR, provided in the assay kit) were applied to anti-mouse EGFR antibody-coated 96-well plates and incubated overnight at 4 °C. After washing the plate with wash buffer provided in the kit, biotin conjugated anti-mouse EGFR antibody was added to the plate and incubated for 1 h at room temperature (RT) with gentle shaking. The plate was then rinsed with wash buffer, treated with horseradish peroxidase-conjugated streptavidin (for 45 min at RT), washed again, and 3,3′,5,5′-tetramethylbenzidine, a peroxidase substrate, was applied for 30 min at RT in the dark. Peroxidase reactions were terminated by adding the Stop solution, and optical absorbance at 450 nm was immediately measured by a 96-well plate reader (Enzo Life Science, Farmingdale, NY, USA). EGFR protein expression (average of duplicate measurements) was normalized by total protein concentration, determined by Bradford assay for each sample.

### 4.5. Laser Doppler Flowmetry

Functional hyperemia was measured by laser Doppler flowmetry as increases in CBF in response to contralateral whisker stimulation, as described previously [8,15,34]. Briefly, under a surgical plane of isoflurane anesthesia, a catheter was inserted in a femoral artery for blood pressure monitoring and a cranial window was prepared over the somatosensory cortex. Cranial windows were superfused with aCSF (aerated with 5% CO_2_/20% O_2_/75% N_2_, warmed to 37 °C). After switching anesthesia to a combination of urethane (750 mg/kg, i.p.) and alpha chloralose (50 mg/kg, i.p.), CBF was monitored via a laser Doppler flow probe (PeriMed, Järfälla, Sweden) placed over the somatosensory cortex through the cranial window. Functional hyperemia was induced by stroking the opposite side vibrissae at a frequency of 3–5 Hz for 1 min (whisker stimulation) and expressed as a percentage increase in CBF. Drugs were applied topically to the somatosensory cortex via aCSF superfusate to the cranial window, except when specifically stated. Whisker stimulation-induced CBF changes were measured three times at each condition (e.g., before and after treatment) in each animal, and the averaged value was used as one data point. Throughout CBF measurements, blood pressure and heart rate were monitored through a femoral arterial cannula, and body temperature was maintained at 37 °C using a heating pad thermostatically controlled by a rectal probe. All data were recorded and analyzed using LabChart 8.1 software (AD Instruments, Sydney, Australia).

### 4.6. Statistical Analysis

Data are presented as mean ± standard error of the mean (*n* = number of the experiments, which, unless noted, also represents the number of animals). Statistical significance was determined by two-tailed Student’s *t*-test for two-group comparisons or by two-way analysis of variance (ANOVA) to compare multiple groups using GraphPad Prism 10 software (GraphPad Software, Boston, MA, USA). *p*-values less than 0.05 were considered statistically significant.

## 5. Conclusions

This study used a newly developed EC-EGFR-KO mouse model to demonstrate that endothelial EGFR contributes to functional hyperemia, an essential CBF regulatory mechanism. In contrast to EGFRs in vascular SMCs, which play a role in arterial contractility, endothelial EGFR signaling modulates focal vasodilatory responses in neuronally active regions of the brain. Disrupting endothelial EGFR signaling impairs functional hyperemia, likely by incapacitating Kir2.1 channel functionality in capillaries. Our study corroborates the previous findings that impaired endothelial EGFR signaling is responsible for functional hyperemia deficits in cSVD/CADASIL model mice. Future studies investigating vascular EGFR signaling, involving both SMCs and ECs, promise to shed additional light on the pathologies associated with cerebral vascular dysfunction occurring in cSVD/CADASIL.

## Figures and Tables

**Table 1:** Physiological parameters in EC-EGFR-KO mice.

	WT (*n* = 8)	EC-EGFR-KO (*n* = 8)
Age (weeks old)	20.2 ± 2.2	20.8 ± 1.3
Body weight (g)	26.8 ± 1.2	27.3 ± 1.4
Blood pressure (mmHg)	91.3 ± 2.9	91.8 ± 1.1
Heart rate (bpm)	498 ± 8	491 ± 6

## Data Availability

Data is contained within the article. The data presented in this study are also available upon request to the corresponding author.
